# Supplementary figures and images for: DNA polymerase theta suppresses mitotic crossing over
Source: PLoS Genet. 2021 Mar 22;17(3):e1009267. doi: 10.1371/journal.pgen.1009267 (PMC8016270; doi:10.1371/journal.pgen.1009267)

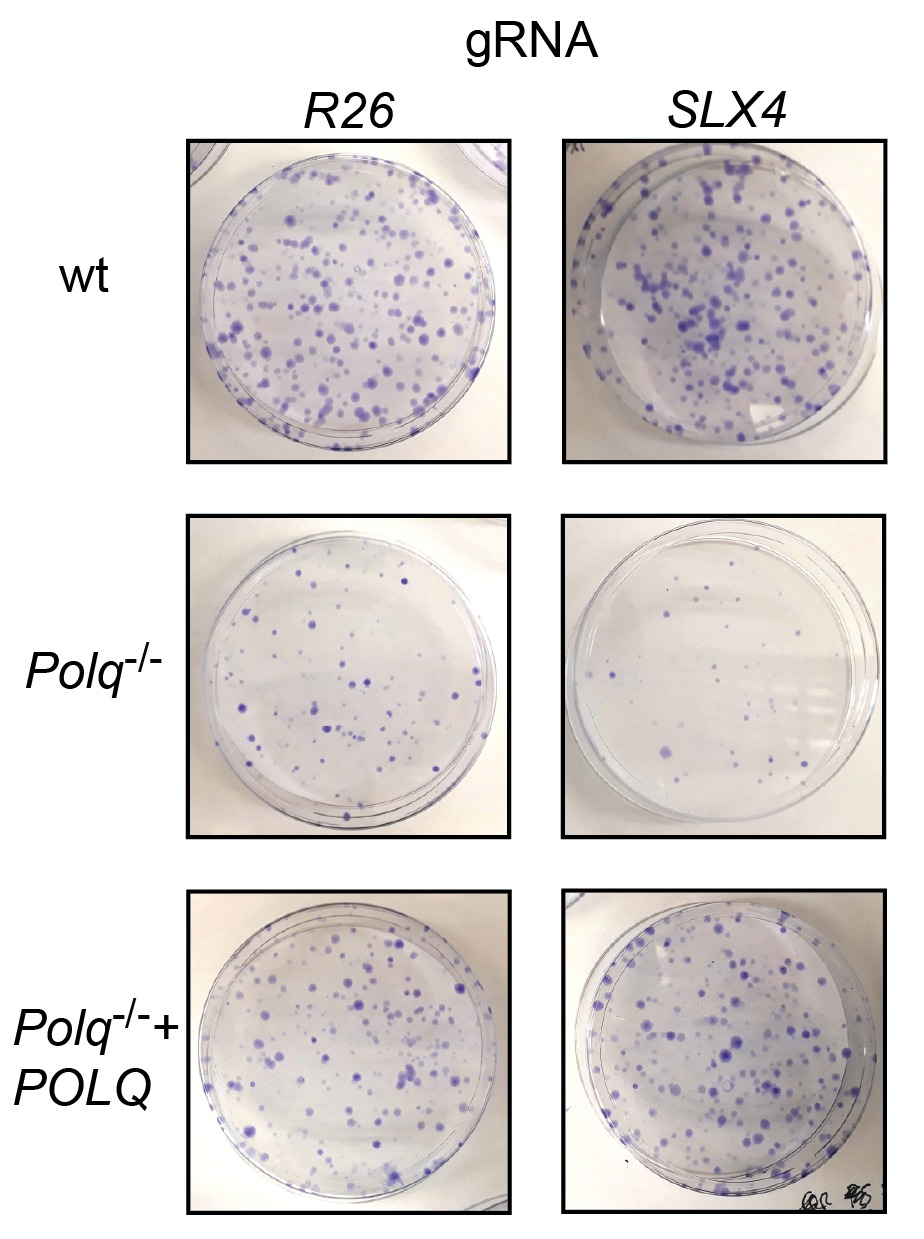

Supplement: S1 Fig — (TIF) [file pgen.1009267.s001.tif]
