# Supplementary material for: DNA polymerase theta suppresses mitotic crossing over
Source: PLoS Genet. 2021 Mar 22;17(3):e1009267. doi: 10.1371/journal.pgen.1009267 (PMC8016270; doi:10.1371/journal.pgen.1009267)
Supplement: S6 Table — (DOCX) [file pgen.1009267.s007.docx]

| **Table S1** | | |
| --- | --- | --- |
| PCR | Primer | Sequence (5’-3’) |
| *PolQ^null^* | Fwd | GGGAACGTGGAGTGTGTACG |
|  | Rev | GAGAAGTACCTGTTCGATGTGC |
| *PolQ^Z2003^* | Fwd | TTATAGTGACGAACGTGCAGC |
|  | Rev | TGCCAATCCTGCTTAGGTTC |
| *mus312^Z1973^* | Fwd | GGCGCTGGCGCTGTCTT |
|  | Rev | CAGGACCGCCAGAGATTTAT |
| *mus312^D1^* | Fwd | TTCTCCTCGAGCTTCTTCTGTGA |
|  | Rev | ACGGTAAGATTGGACTTACG |
| *Gen^Z5997^* | Fwd | TGCAGTGGCCAGAAATGC |
|  | Rev | AGCATGTCCAATGGCTCAAT |
| *Df(3L)6103* | Fwd | TTTACTCCAGTCACAGCTTTG |
|  | Rev | CACTTTGGTCGTTAGTCCTGGTG |
| *slx1^F93I^* | Fwd | ATACAGTGGCACTCCAAA |
|  | Rev | TCTCGTAATCCGTTTCTAGC |
| *slx1^e01051^* | Fwd | AATGATTCGCAGTGGAAGGCT |
|  | Rev | TTAGAGGGGCAGTAAGTACAGTAAGTA |
| *ry* cut site | Fwd | CTGGGTTCTTCACTGGCTATC |
|  | Rev | AGTTCGAGGAGCGAGCACT |
| *R62* cut site | Fwd | GGCGGATCACAAGCAATAAT |
|  | Rev | TCAGTTGGGCTGTTTTGGAG |
| *SLX4* cut site | Fwd | TGCTGCTGGTTTTAGTTTGCCTG |
|  | Rev | AACCCAAGTGTCTGGCTATG |
