# Supplementary material for: DNA polymerase theta suppresses mitotic crossing over
Source: PLoS Genet. 2021 Mar 22;17(3):e1009267. doi: 10.1371/journal.pgen.1009267 (PMC8016270; doi:10.1371/journal.pgen.1009267)
Supplement: Attachment — Submitted filename: Response to reviewers.docx [file pgen.1009267.s008.docx]

**Comments to the Authors:**

**Please note here if the review is uploaded as an attachment.**

We wrote individual responses to each point raised by the reviewers in blue font.

**Reviewer #1**: This manuscripts requires very little commenting by me. The abstract provides a accurate summary of the data presented and the novelty of the study: extending our knowledge on the synthetic lethal interaction between Pol theta on the on hand, and homologous recombination on the other. The paper is very concise but makes the point on shared intermediates between TMEJ and Holliday junction formation/resolution quite clearly and convincingly by a set of genetic experiments making use of an elaborate set of single, double and triple mutant flies . The manuscript is well written in the sense that it was easy to read (not always the situation for Drosophila papers). It is also appreciated that the genetic interaction as validated in mammalian cells. The notion that TMEJ suppresses mitotic crossing over, hence preserving genomic stability (at the expense of indels) is interesting.

I have only two small comments:

i) in some instances, the wording "share a common substrate" may give the impression an incorrect impression, while I realise it is not what the authors suggest. For instance, the scenario that TMEJ can process unresolved HR intermediates that may manifest in SLX4 mutants does not mean that HR intermediates are intrinsic to TMEJ (page 9 line 189/190). I realise that the sentence could be read such that the common DNA substrate is DSBs with 3' ssDNA tails but it is nevertheless a bit confusing.

First, we’d like to thank Reviewer 1 for their positive review.

Regarding the first minor point: In lines 189/190, we agree that “suggests that their repair pathways share a common DNA substrate” may be confusing. As the reviewer says, the substrate we mean is not 3’ resected ends, but it may be interpreted that way. We substituted that sentence with “suggests the existence of a DNA intermediate that will either be joined by TMEJ or progress to a double Holliday junction and be resolved by SLX4 or GEN1”.

As part of our response to Reviewer 3, we added the following paragraph to the discussion, which also helps clarifying this point: “SDSA is upstream of TMEJ and Holliday junction formation/resolution, yet both Pol θ-associated indels and mitotic crossovers are observed in wild type flies. This indicates that sometimes SDSA either fails or cannot be completed. We propose that the leftover DNA intermediate(s) can either be joined by Pol θ, generating a small indel, or can progress to a double Holliday junction, that will be resolved forming a mitotic crossover.”

ii) The idea that TMEJ can act on HR intermediates post D-loop invasion was recently reported in worms (Kamp et al., Nat. commun. 2020). Include this study as it seems highly relevant.

We now cite this work in lines 116-119: “However, DNA intermediates formed downstream of end resection and strand invasion may also be amenable for repair by TMEJ. This has recently been suggested to be the case when long-range resection is impaired due to mutations in *BRCA1*, which may inhibit the re-annealing of the unwound D-loop [22].”

**Reviewer #2**: This is an interesting investigation into the role of polymerase theta in DNA repair and its relationship with other proteins involved in homologous and nonhomologous repair of DNA double strand breaks. The results would be a useful addition to the literature except for, primarily, one significant problem. That problem relates to data presentation, and the fact that, as presented, the results are presented only after the authors have analyzed them as they wish - the raw data are nowhere to be found. This makes it quite difficult to judge the validity of the authors' conclusions, and it does not allow readers (or reviewers) to consider alternative interpretations. This deficiency must be remedied. The presentation of results would be fine for a seminar, but not for a publication.

Detailed critiques below:

line 108 - should be Humeral (not Humoral). This marker, and Curly, are presumably on balancers. Please specify which chromosomes are being used here, and throughout. In all cases the actual crosses should be given. This is an even more significant issue in later experiments.

We have added the balancers used in the experiments related to Figs 1b and 1c, in lines 106-110: “Homozygous mutant flies are easily identified due to the presence of a homologous balancer chromosome (*CyO*, *Cy^1^ dp^lvl^ pr^1^ cn^2^* on the second chromosome and *TM6B,* *Antp^Hu^ Tb^1^ e^1^ ca^1^* on the third chromosome) that contain a dominant phenotypic marker (*Curly* (*Cy*) for *BRCA2*, *Humeral* (*Antp^Hu^*) for *POLQ*) (Fig 1B).” We have also added the balancers to Figure 1b.

We thank Reviewer 2 for the very careful assessment of our work, which allows us to correct typos such as “*Humoral*”.

line 127 - present detailed results please, not just a summary. In the double mutant cross, were single mutant viabilities scored, and did they confirm results from previous two crosses? Or was there possibly some dominant interaction?

We now include the raw data in table S1 (total of balanced (heterozygous) and unbalanced (homozygous) flies), with the % observed and expected homozygous mutant flies.

*SLX4* *GEN1* and *POLQ* are on the same chromosome (third), so we cannot look at the single mutants in these crosses. To clarify this, we added the following sentence to the figure legend: “Since *POLQ*, *SLX4*, and *GEN1* are on the third chromosome, the balancer *TM6B,* *Antp^Hu^ Tb^1^ e^1^ ca^1^* was used in these crosses.”

line 171 - Expression of Cas9 is causing damage - so this experiment is not done "in the absence of exogenous damage". It would be useful to confirm this result by another method, perhaps using RNAi to knock down SLX4.

We have added “except for the DSB made by Cas9” (lines 176) to this sentence. We now also note in methods the control guide we used also introduces a DSB – indeed, this guide is actually more efficient at editing, though at a “safe harbor” locus, as now reported in the method section – indicating the impact of *SLX4* editing on relative survival is not explained by effects of chromosome breakage by Cas9.

RNAi is typically not used for this type of experiments, because the knock down in mammalian cell culture is often not sufficiently durable for the 10 days required to visualize colonies.

We note *SLX4* similarly came up in a CRIRPR screen for synthetic growth defects with *POLQ* (Feng et al., 2019. PMID: 31537809) that used 10 guides/gene, none of which were the one used in this study, arguing against off-target effects being the cause for the observed lethality.

line 174 and experiment that follows - the cuticular defect mentioned is properly referred to as "etched tergites". Unfortunately, the photo is a very poor representation of what, I imagine, the authors observed. First, the photos have an extreme yellow tint, making it difficult to see the cuticle; second, the arrow seems to rest right on top of the disturbed cuticle; third, there is no quantitation of this defect, making it impossible to judge its significance. Since this study examines apoptosis in wings, were there any wing patterning defects?

We now use the term “etched tergites” to describe our observations. Also, we took, and now include, better pictures of the fly abdomens, as we eliminated the yellow tint that the reviewer mentions. In addition, we have quantified this phenomenon in wt, *POLQ*, *SLX4*, and *POLQ* *SLX4* mutants. (lines 180-183): “Interestingly, etched tergites (disrupted tissue patterning in the abdomen) could be readily observed in most *POLQ* *SLX4* double mutant flies (88.1%, n=42) (Fig 4A). These are indicative of defects in cell survival or proliferation during development. We never observed them in wt (n=71) and *POLQ* mutants (n=40) and rarely in the *SLX4* ones (18.2%, n=44).”

No wing defects were observed in the *POLQ* *SLX4* mutant flies. We explain this by the ability of wing discs to perform compensatory proliferation to make up for the dead cells.

line 185 (and 246) - the authors state that the viability reduction of the POLQ SLX4 Gen1 triple mutant is "due to endogenous DNA damage". This has certainly not been demonstrated. It seems likely that this is true, but it has not been shown that these proteins have no roles outside of DNA repair. The authors should slightly temper this statement.

We removed the sentence in line 185 claiming that the viability reduction of the *POLQ* *SLX4* *GEN1* triple mutant is "due to endogenous DNA damage". For line 246 (now 256), we added that the high levels of apoptosis are “likely” caused by endogenous DNA damage.

line 198 (experiment described starting here) - this experiment definitely needs a more complete and detailed description. What were the precise crosses? Did Cas9 come maternally or paternally? (Since its driven by nanos, this matters.) What were maternal and paternal genotypes with respect to mutants being analyzed? These details must be shown - the results cannot be fully evaluated without them.

To make the experiment clearer, we now include, in the methods section (lines 365 and 382-387), the crosses that lead to the males used in the experiments described in Figure 5. These crosses show that Cas9 comes maternally.

line 219 - "was required to" completely "eliminate mitotic crossing over" (add for clarity)

line 220 - "is" in "contrast to..." (grammar)

We thank the reviewer for helping us fix these grammar issues.

Table 1 - needs a legend explaining details of experiment

We now include a table 1 legend, below the figure legends.

lines 300, 301 - use of term "heteroallelic mutant flies" in line 300 and "homozygous mutant flies" on line 301, in the same sentence, is confusing. I believe the authors are referring to the same genotype in both cases.

We agree that this can be confusing and we have fixed it.

line 326 - "Heads of third instar larvae..." Do larvae have heads?

We now indicate that “The anterior halves of third instar larvae…”

line 327 - "inverted"? Please elaborate.

We changed the word “inverted” for “everted”, which better describes the process.

line 340 - "these flies were heterozygous" for "the genes..." (grammar)

This was fixed.

line 352 - "single males as the ones described above..." Which males?? Clarify please. Showing crosses would help with this.

We now show the crosses (lines 382-387).

Figure 2 - Each dot represents a vial pair? Please give more detail in legend or in Methods. Also, this method of presenting results, as a ratio of treated to untreated, obscures the underlying data. The actual results obtained in the experiment should be presented, not just this abstract graphical treatment. Also, which differences are significant?

We now include that each dot represents a vial pair in the figure legend.

We now have a Table S2 which has the raw data for the experiments in figs 2B, 2C and 2D.

Figure 3 - Reporting only relative survival obscures absolute survival numbers (i.e., overall impact of cutting, replating). Does Cas9 cutting affect survival of cells, particularly polq mutant cells?

Each cell line has a different plating efficiency (this is a common phenomenon for mammalian cell culture), so absolute survival numbers are uninformative. The raw data is now included in Table S3. Additionally, as now noted in the methods, cutting efficiency for the R26 guide is approximately two fold higher than observed for the SLX4 guide, yet there is no difference in colony numbers in either wild type or complemented cell line backgrounds, arguing against a significant impact of cutting efficiency on colony forming ability.

We did not test whether Cas9 has an effect in survival of cells.

Figure 4 - part A needs a better photo (and quantitation); part B - these discs do not appear to be at the same stage of development - could that impact the results?

It is unclear to us whether the differences in disc morphology is due to the high apoptosis levels (especially for the triple mutant) or if there is a developmental difference between the genotypes. All larvae dissected were the same chronological age (5-7-day old) and there wasn’t an apparent disc size difference. The developmental question in an interesting one (beyond the scope of this study). Regardless, we do not think that this could impact the results of this figure.

Figure 5 - This experiment really needs to show the crosses used. In part A - were both chromosomes ry+ to start? I believe this to be the case, but the Figure makes it confusing because it labels the mutant alleles (st and e) in exactly the same way as the wildtype allele (ry, no +). Part D - It is not entirely clear what we're looking at here: is this % of progeny that are recombinants, or % of fathers that produced any recombinants? Neither the explanation in the legend, or in the text, or in Methods is clear enough to know the answer to this with certainty. I also have other questions: Were reciprocal crossovers recovered equally? Were crossovers ever associated with the occurrence of ry mutants? If so, which recombinant were they on? Finally, how many males were tested? (Answer in Table 1?) How many progeny were scored?

Crosses are now shown in the methods. We agree that the figure makes it look like the maternal chromosome is ry mutant, and the reviewer is right, it is not. We have removed the gene from the figure.

Figure 5d represents the % of progeny that are recombinant (figure legend has been changed to makes this clear).

Reciprocal crossovers were not always recovered equally.

We never tested whether the crossovers were associated with *ry* mutations. Anecdotally, we sequenced a couple of recombinant flies and found no deletions associated with the crossover event. However, the number of flies sequenced is not high enough to be meaningful.

The number of males tested for each genotype is indicated in the figure legend. We now also include the total number of progeny scored.

In sum, this paper appears to represent significant and interesting work. When the flawed presentation of data is remedied, it should be reconsidered.

**Reviewer #3:** This is paper from the Sekelsky Lab reports a synthetic lethality between POLQ and the HJ resolvases SLX4 and GEN1 in fruit fly. While it was previously known that TMEJ repair via POLQ is necessary for survival of BRCA1/2 mutant cells, a synthetic genetic interaction with genes involved in later steps of HR was not previously demonstrated. The authors expand their finding to mammals and show that in mouse embryonic fibroblasts, the lack of POLQ and SLX4 result in a strong genetic interaction. Back to flies, the authors observe drastic increase in apoptosis in cells lacking POLQ, SLX4 and GEN1. Of importance, POLQ depletion in flies leads to 18-fold increase in mitotic crossovers after Cas9-induced chromosome break, consistent with the model that in the absence of POLQ, repair intermediates are directed to HR repair, HJ formation and the action of SLX4 and/or GEN1.

Overall, this is a well structured, well written and straightforward manuscript. The reported findings are novel and should have an important impact in the DNA repair community. The major point of improvement is related to the proposed model for how the lack of POLQ results in the increased requirement for resolvase action. The authors don't provide a clear model and don't elaborate much on this key part of the paper. The authors should consider the possibility that altered resection dynamics upon lack of POLQ may be triggering the increase in HJ formation (see below). In addition, the results in the paper also beg the question as the whether POLQ is also required upon lack of BLM.

Major points

1. The authors mention that "TMEJ and Holliday junction formation/resolution share a common substrate” but fail to clarify/discuss which substrate this may be. The authors should consider that changes in DNA end resection in the absence of POLQ may be causing the requirement for the resolvases. In the absence of POLQ, DNA ends with short resected tracts, which would mostly be repaired by TMEJ, may have more opportunity to be further ressected, leading to accumulation of ends with longer resected tracks that may become the substrate for canonical HR and the formation of more HJs. The authors mention that SDSA frequency is not altered by lack of POLQ, and seem to hastily conclude that POLQ does not compete for the 3’ ends generated by 5’ end-resection. However, this does not seem a compelling enough argument to prove that there are not more ends channeling to canonical HR and HJ formation. It would be useful if the authors are able to monitor resection (preferably using End-Seq) and assess whether more ends are resected, and longer resected tracks are generated upon lack of POLQ.

In the response to Reviewer 1, we have clarified what we mean by “TMEJ and Holliday junction formation/resolution share a common substrate”, which now reads: “The strong genetic interaction between Pol θ and the resolvases suggests the existence of a DNA intermediate that will either be joined by TMEJ or progress to a double Holliday junction and be resolved by SLX4 or GEN1” in the results section (lines 196-198).

We have also added a couple of sentences to the discussion: “SDSA is upstream of TMEJ and Holliday junction formation/resolution, yet both Pol θ-associated indels and mitotic crossovers are observed in wild-type flies. This indicates that sometimes SDSA either fails or cannot be completed. We propose that the leftover DNA intermediate(s) can either be joined by Pol θ, generating a small indel, or can progress to a double Holliday junction, that will be resolved forming a mitotic crossover,” to clarify our proposal for a DNA substrate shared by these two pathways. This is an important point of our paper, and we are glad Reviewer 3 asked us about it, so that it could be clearer.

Regarding the possibility of TMEJ utilizing a differently resected DNA substrate “short resected tracts”, there is no evidence of this phenomenon in wild type-flies or cells, even though it has been suggested to be of importance in the absence of *BRCA1*. As the reviewer mentions, SDSA is not increased in the absence of Pol θ in flies, which argues against Pol θ acting on short resected ends before larger resected ends can be generated. Furthermore, this is also true for SSA, another pathway that requires long resection, as it is not increased in *POLQ* mutants in flies (Wei and Rong, 2007, PMID: 17660539) and human cells (Kelso et al., 2019, PMID: 31381562) (we now cite these papers in the discussion, lines 270-272).

In addition, *POLQ* KD also doesn’t increase SSA or HR in *Drosophila* cells in culture. In the case of HR, it was measured after a Cas9 break and by using a linear or circular, exogenous DNA template (Schmidts et al., 2016. PMID: 27353331).

We agree that the End-seq is the best method available to monitor the length of end-resection tracts. However, we consider that adapting this technique to *Drosophila*, which would involve switching Cas9 expression to be rapidly inducible, to be beyond the scope of this project.

The editor suggested measuring gene conversion tracks in HR events after cutting by Cas9. Gene conversion tract lengths are a combination of resection, synthesis length, and mismatch repair, so we do not think we could make strong conclusions about resection length from tract length. For example, one can imagine long resection leading to short synthesis off the homolog (with the remainder filled in after annealing) to make a short conversion tract; or short resection followed to long synthesis plus strand displacement and MMR after annealing, to create a long conversion tract.

2. Taken together, the work does beg the question as to whether POLQ deletion is synthetic lethal with BLM deletion. Even if SDSA frequency is not altered by lack of POLQ, one wonders if the role of BLM in promoting dissolution would become essential in the absence of POLQ. In fact, an interesting prediction is that in the absence of BLM, POLQ could play an even more important role in limiting drastic increases in mitotic crossing overs. Exploration of the POLQ and BLM genetic interaction could significantly increase the impact of the paper and further strengthen the central claim that POLQ suppresses mitotic crossing over.

We agree that testing whether *POLQ* and *BLM* are synthetic lethal may be of interest. However, BLM’s multiple roles in HR (end-resection, SDSA, and dHJ dissolution) would make interpretation of the results ambiguous, requiring the development of additional assays and/or separation-of-function BLM mutants that are significantly more sophisticated than currently available. While undoubtably interesting, we consider it is beyond the scope of this project.

Minor points

I. On Figure 3, it would be reassuring if the authors could show a western blot confirming that the editing of the SLX4 gene impacted SLX4 protein levels, as well as show representative images for the colony formation survival assay.

We agree that it would be good to have a western blot. However, we could not get an antibody to reliably detect SLX4 in mouse cells.

We now include representative pictures of the plates in Figure S1.

II. For most presented data in the paper, including survival data from flies or mouse cells, please show p-value whenever appropriate. I understand that p-values are not always essential/relevant, but for cases where the authors think p-value are not necessary, I would like a quick explanation as for why.

We now include statistical significance information for Figures 2b-d, 3b, 4c, and 5d. For Figures 1b/c and 2a, we consider the differences in survival to be large enough that a statistical test is not necessary

III. For Figure 4A, besides the representative image, it would be useful to show the number of POLQ and SLX4 mutant female flies that presented the defects in abdominal banding pattern and to discuss a little more of how POLQ and SLX4 could be related to this developmental .

We now include quantification of this phenomenon (lines 180-183). We also added that this is indicative of defects in cell survival or proliferation during development (better showed by the increased apoptosis, fig 4c). We do not think *POLQ* and *SLX4* are involved in development *per se*, but increased cell death during development due to DNA damage will lead to developmental defects like this one.
